# Investigating the Therapeutic Mechanisms of Shen-Ling-Bai-Zhu-San in Type 2 Diabetes and Ulcerative Colitis Comorbidity: A Network Pharmacology and Molecular Simulation Approach

**DOI:** 10.3390/ph18101516

**Published:** 2025-10-10

**Authors:** Qian Yu, Shijie Sun, Tao Han, Haishui Li, Fan Yao, Dongsheng Zong, Zuojing Li

**Affiliations:** Department of Pharmaceutical Informatics, Shenyang Pharmaceutical University, No. 103 Wenhua Road, Shenyang 110016, China; qianq200129@outlook.com (Q.Y.); sunshijie1222@163.com (S.S.); 18048970825@163.com (T.H.); haishui755377@163.com (H.L.); 13050264813@163.com (F.Y.)

**Keywords:** type 2 diabetes, ulcerative colitis, molecular dynamics simulation, network pharmacology, bioinformatics, flavonoids

## Abstract

**Objectives**: Shen-Ling-Bai-Zhu-San (SLBZS) is a classical traditional Chinese herbal formula with spleen-invigorating and dampness-resolving properties. Recent pharmacological studies suggest its potential to regulate immune and metabolic disorders. Type 2 diabetes mellitus (T2D) and ulcerative colitis (UC) often coexist as comorbidities characterized by chronic inflammation, microbial imbalance, and insulin dysregulation, yet effective therapies remain limited. This study aimed to investigate the molecular mechanisms through which SLBZS may benefit T2D–UC comorbidity. **Methods**: An integrative multi-omics strategy was applied, combining network pharmacology, structural bioinformatics, and ensemble molecular docking–dynamics simulations. These complementary approaches were used to identify SLBZS bioactive compounds, predict their putative targets, and examine their interactions with disease-related biological networks. **Results**: The analyses revealed that flavonoids in SLBZS act on the SLC6A14/PI3K–AKT signaling axis, thereby modulating immune responses and improving insulin sensitivity. In addition, SLBZS was predicted to regulate the NF-κB/MAPK signaling pathways, key hubs linking inflammation and metabolic dysfunction in T2D–UC. These dual actions suggest that SLBZS can intervene in both inflammatory and metabolic processes. **Conclusions**: SLBZS demonstrates promising therapeutic potential for T2D–UC by targeting interconnected immune–metabolic networks. These findings not only provide mechanistic insights bridging traditional therapeutic concepts with modern pharmacology but also establish a theoretical basis for future experimental validation and clinical application.

## 1. Introduction

Type 2 diabetes (T2D) is a chronic metabolic disorder characterized by persistent hyperglycemia due to impaired insulin secretion or action. Its prevalence is rising worldwide [1]. Between 1990 and 2019, diabetes-related deaths increased substantially in China (from 64,084 to 168,388) and globally (from 606,407 to 1,472,934) [2]. More than half of T2D patients develop complications, making it a leading cause of disability and mortality [3].

Inflammatory bowel disease (IBD), which includes ulcerative colitis (UC) and Crohn’s disease (CD), also affects approximately 0.3% of the global population [4]. UC is characterized by chronic mucosal inflammation of the colon, driven by immune dysregulation and microbial imbalance [5,6].

T2D and UC frequently coexist and share pathophysiological mechanisms such as genetic susceptibility, immune dysfunction, and gut microbiota disruption [7,8,9]. Large-scale prospective evidence supports this overlap: in a UK Biobank cohort of 313,008 participants, T2D was associated with higher risks of IBD (HR 1.61), Crohn’s disease (HR 2.10), and UC (HR 1.58) compared with non-diabetic individuals [10]. Diabetes is one of the most frequent comorbidities in UC, and their association suggests shared genetic and clinical pathways. The coexistence of T2D and UC is linked to overlapping complications such as neuropathy, hepatic steatosis, osteoporosis, and venous thrombosis. Moreover, hyperglycemia increases surgical risks, including postoperative complications and pouch failure. Management is further complicated by corticosteroids, a mainstay of UC treatment, which can worsen glucose intolerance [11]. These challenges underscore the need for preventive measures and new therapeutic strategies.

Dietary interventions may offer one approach. A case study reported that a plant-based diet, combined with diabetes medication, led to UC remission and improved glycemic control in a diabetic patient [12]. Similarly, antidiabetic drugs such as metformin and empagliflozin have shown anti-inflammatory effects in colitis models [13], suggesting potential benefits for both diseases. However, effective early treatments remain limited.

Given these gaps, traditional remedies such as Shen-Ling-Bai-Zhu-San (SLBZS) warrant investigation. SLBZS is a classical herbal formula widely used in traditional Chinese medicine (TCM). Clinical studies show that SLBZS, combined with Erchen Decoction and acupuncture, improves insulin resistance and glycemic indices more effectively than conventional therapy alone [14]. In UC, SLBZS combined with mesalamine enhances mucosal healing and alleviates colon injury, partly by modulating gut microbiota and microbial tryptophan metabolites [15]. Although evidence supports its benefits in T2D or UC separately [16], its role in comorbid T2D–UC has not been systematically studied.

**Figure 1:**
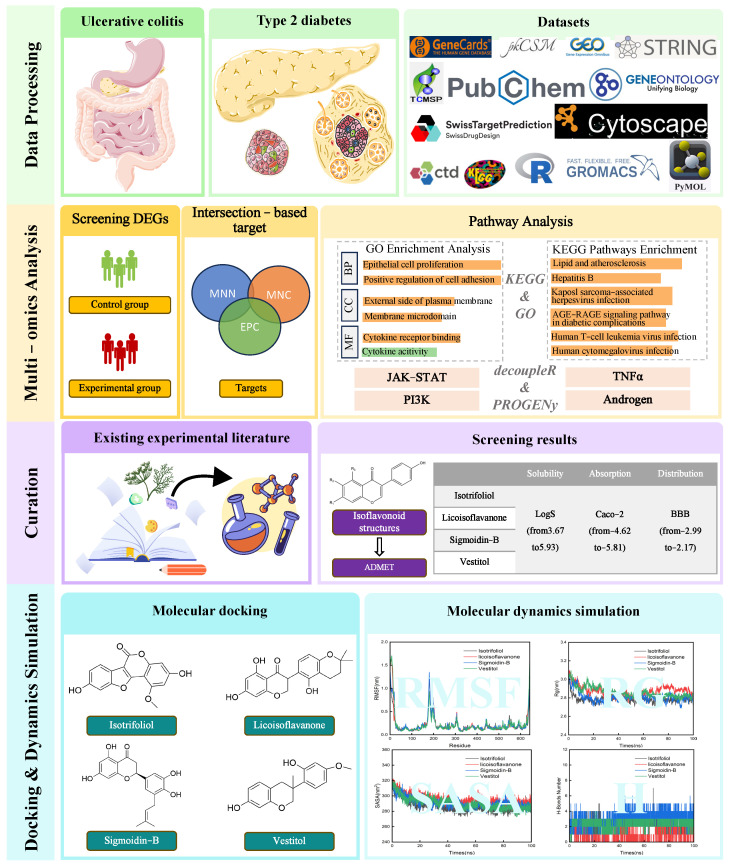
Flowchart outlining the research approach to investigate the therapeutic mechanisms of Shen-Ling-Bai-Zhu-San in the comorbidity of type 2 diabetes and ulcerative colitis.

This study investigates the molecular mechanisms and therapeutic potential of SLBZS in T2D–UC using network pharmacology, molecular docking, and molecular dynamics simulations to identify bioactive compounds and shared targets. Our goal is to provide insights into dual-target therapies for these comorbid diseases by integrating traditional medicine with modern pharmacology. The workflow is illustrated in Figure 1.

## 2. Results

### 2.1. Screening of SLBZS and Disease-Related Targets

**Figure 2:**
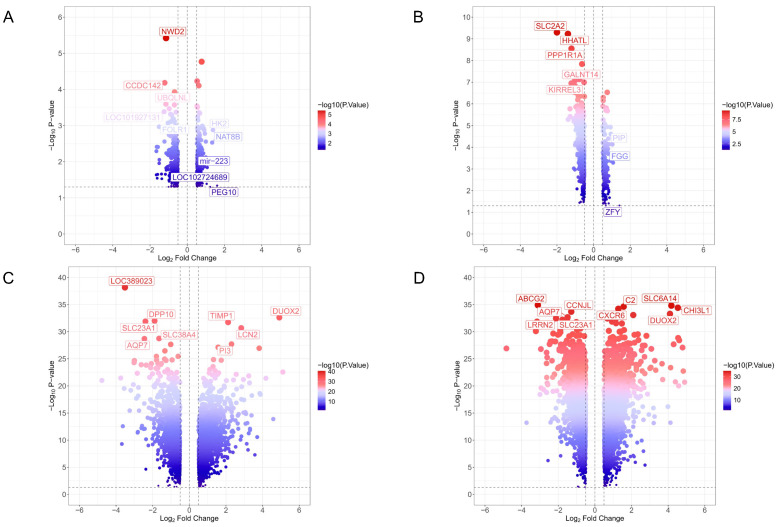
Volcano plot of (**A**) GSE23343, (**B**) GSE76894, (**C**) GSE87466, and (**D**) GSE107499 datasets. In the volcano map, blue color indicates the downregulated genes, while red color represents the upregulated genes.

**Figure 3:**
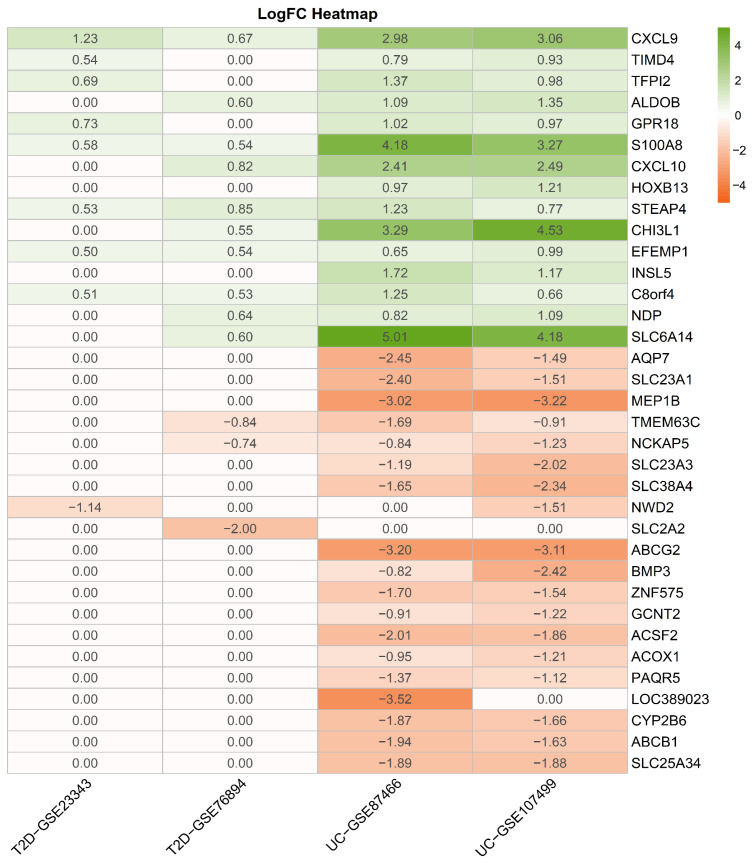
Common differentially expressed genes in the comorbidity from GEO datasets, including 13 upregulated and 3 downregulated genes.

Initially, differential expression analyses were performed on four GEO datasets related to the diseases, using the thresholds $|\log_{2}\;\mathrm{fold}\;\mathrm{change}|>0.5$ and adjusted *p*-value <0.05. The identified DEGs are summarized in Figure 2. After merging and filtering across datasets, 13 genes were found to be consistently upregulated in both ulcerative colitis and type 2 diabetes, with CXCL9, S100A8, CXCL10, CHI3L1, and SLC6A14 showing the most significant alterations. Moreover, three genes—TMEM63C, NCKAP5, and NWD2—were consistently downregulated (Figure 3).

**Figure 4:**
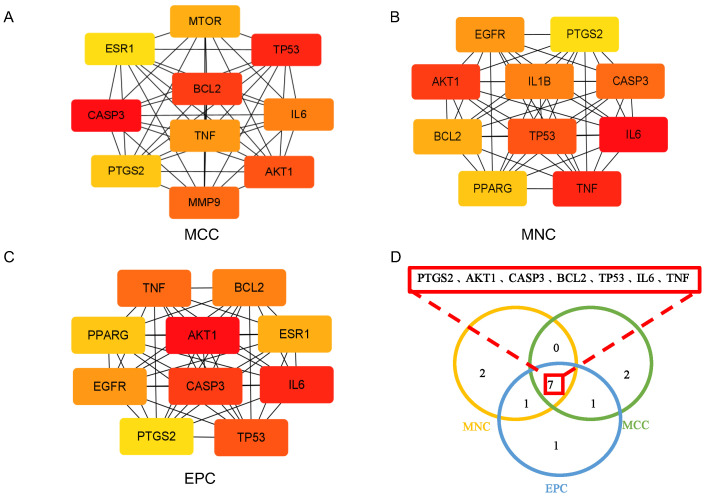
Analysis of common targets related to SLBZS and comorbidity of ulcerative colitis and type 2 diabetes. (**A**–**C**) Network analysis of shared targets between SLBZS and the comorbidity using Cytoscape. Top 10 targets were identified by (**A**) MCC, (**B**) MNC, and (**C**) EPC methods. (**D**) Seven key targets were selected from the intersection.

For potential therapeutic target identification, preliminary screening of active compounds in SLBZS yielded 6045 predicted targets. Among these, 2097 were associated with ulcerative colitis and 6459 with type 2 diabetes, with 804 overlapping between the two diseases. To further prioritize, the intersection of SLBZS-related and disease-related targets was imported into Cytoscape 3.9.1 for network analysis. Topological evaluation was conducted using MNC, MCC, and EPC methods, and the top ten targets from each were extracted. Their intersection yielded seven core targets, which were considered key common targets (Figure 4A–D).

### 2.2. Pharmacokinetic Profiling of Bioactive Constituents

**Figure 5:**
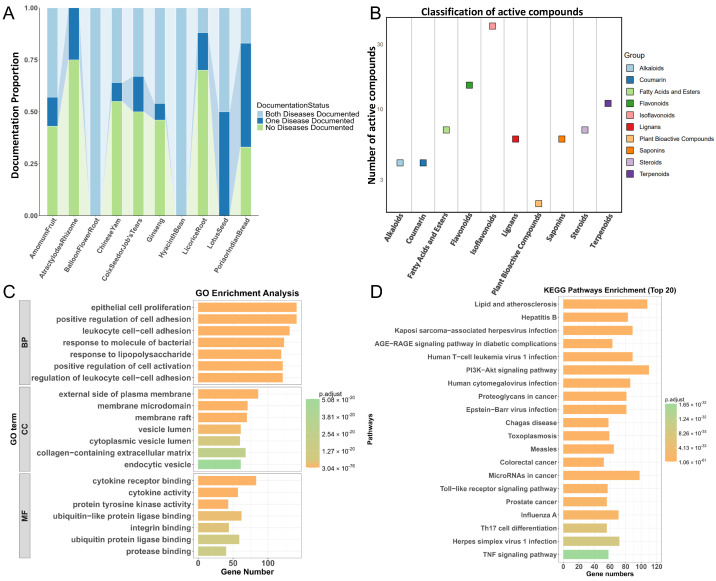
Systematic curation of pharmacologically active small molecules in SLBZS. (**A**) Hierarchical analysis of herbal components in evidence-based traditional Chinese medicine formulas, (**B**) Distribution of compound proportions in traditional Chinese medicine formulas lacking experimental evidence. Functional enrichment analysis. (**C**) GO Enrichment Analysis, (**D**) KEGG Enrichment Analysis.

A systematic curation of the pharmacologically active small molecules of SLBZS was carried out by analyzing the evidence-based literature, stratifying herb-derived compounds by their experimentally validated therapeutic functions in the treatment of T2D, UC or dual diseases versus unvalidated candidates (Figure 5A). Among 103 nonvalidated compounds, flavonoid derivatives constituted the predominant class (n = 41, 39.8%), warranting a prioritized pharmacokinetic evaluation (Figure 5B).

Subsequent ADMET analysis using the pkCSM platform identified four flavonoid leads—Isotrifoliol, Licoisoflavanone, Sigmoidin-B, and Vestitol—with favorable drug-likeness properties in accordance with Lipinski’s criteria (molecular weights: 272.30–356.37 Da; ≤3 rotatable bonds; hydrogen bond acceptors: 4–6; hydrogen bond donors: 2–4; LogP: 2.825–3.724). The main pharmacokinetic and safety-related findings are summarized as follows (Table 1):

- Solubility: Predicted LogS values ranged from −3.67 to −5.93, consistent with adequate solubility across physiological conditions.
- Absorption: Caco-2 permeability (−4.62 to −5.81 logPaap) and high human intestinal absorption (88.1–99.8%) indicated strong potential for oral bioavailability.
- Distribution: All compounds showed limited blood–brain barrier penetration (logBB: −2.99 to −2.17) and moderate volume of distribution (VDss: 0.88–1.58 L/kg), suggesting restricted CNS exposure and balanced tissue distribution.
- Metabolism: The compounds were predicted to be non-substrates of CYP3A4 and showed no inhibition of P-glycoprotein, suggesting favorable metabolic stability and a potentially lower risk of efflux-mediated resistance.
- Toxicity: Low hepatotoxicity potential was observed, with drug-induced liver injury (DILI) probabilities ranging from 23% to 31%.

### 2.3. Functional Enrichment Analysis

#### 2.3.1. Gene Ontology (GO) Enrichment Analysis

GO enrichment analysis of prioritized targets identified 4160 significant terms, including 3735 biological processes (BP), 276 molecular functions (MF), and 149 cellular components (CC). Key BP terms were related to inflammatory regulation (e.g., cytokine signaling and leukocyte activation), epithelial repair (cell proliferation and integrin-mediated adhesion), and bacterial responses, suggesting a mechanistic link between immune dysregulation and mucosal barrier dysfunction in T2D-UC comorbidity. MF enrichment highlighted cytokine receptor binding and extracellular matrix interactions, whereas CC enrichment emphasized membrane raft organization and apical junction complexes, indicating that SLBZS may regulate multiple cellular compartments (Figure 5C).

#### 2.3.2. Kyoto Encyclopedia of Genes and Genomes (KEGG) Enrichment Analysis

KEGG pathway analysis identified 186 enriched cascades. The AGE-RAGE, TNF, and Toll-like receptor (TLR) pathways emerged as major drivers of chronic inflammation and immune overactivation in disease progression. Th17 cell differentiation and PI3K-Akt/MAPK signaling were key nodes linking metabolic disturbances (e.g., insulin resistance) to impaired epithelial repair. Enrichment of lipid metabolism and atherosclerosis pathways indicated cardiovascular comorbidity risks, while apoptosis and colorectal cancer pathways suggested oncogenic potential in T2D-UC comorbidity. These findings highlight SLBZS as a multi-target therapeutic agent that may promote inflammatory resolution, restore metabolic homeostasis and epithelial integrity, and mitigate vascular and carcinogenic complications (Figure 5D).

### 2.4. Core Pathway Prioritization and Gene Regulatory Mapping

**Figure 6:**
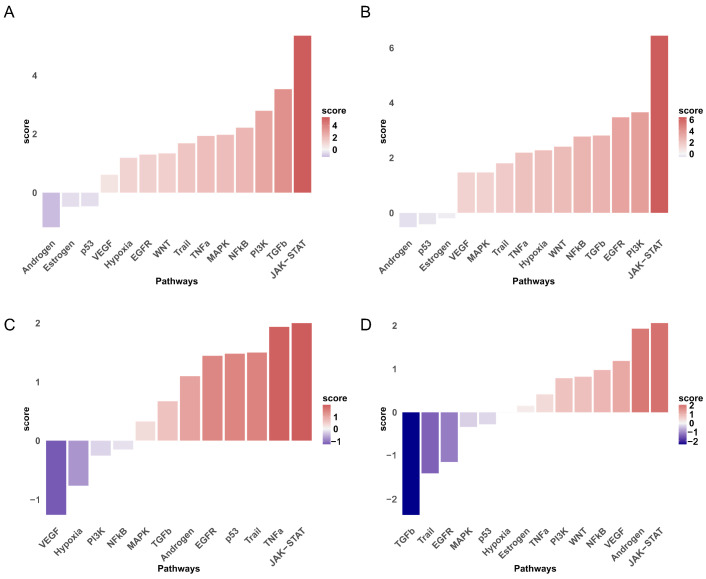
Ranking of pathway activity in the four GEO datasets: (**A**) GSE107499, (**B**) GSE87466, (**C**) GSE23343, and (**D**) GSE76894.

Pathway activity profiling with the decoupleR framework delineated signaling networks underlying T2D-UC comorbidity. After preprocessing (missing value imputation, quantile normalization, and log_2_ transformation), the PROGENy human pathway atlas was queried to construct a topology-weighted network of 500 disease-relevant pathways. Multivariate linear modeling (MLM) with sample-wise activity scores across four GEO datasets identified JAK-STAT, PI3K, TNF-α, and androgen receptor signaling as top-ranked pathways mediating inflammatory–metabolic crosstalk (Figure 6A–D).

**Figure 7:**
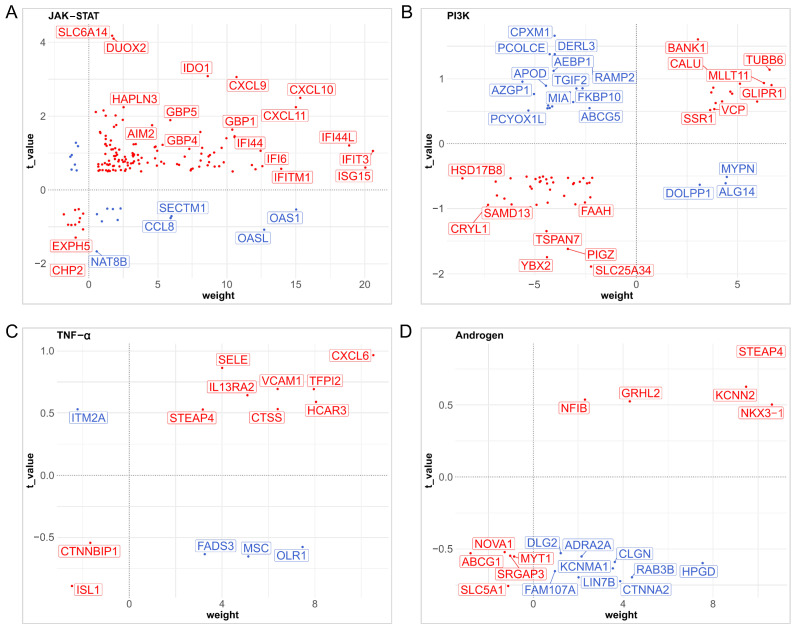
Specific distribution of genes and interaction relationships (**A**–**D**).

Pathway activity gradients showed condition-specific gene expression. Weighted z-score heatmaps highlighted key upregulated regulators (e.g., STAT3, PIK3CA, and TNFRSF1A) and downregulated regulators (e.g., AR, SOCS3, and PTEN) across disease states (Figure 7A–D). Mapping of gene–pathway interactions indicated clustered enrichment of inflammatory mediators (CXCL9/10 and IL6ST) within the TNF-α/JAK-STAT axes, whereas metabolic disruptors (IRS1 and AKT2) localized to PI3K nodes. Together, these findings elucidate coordinated transcriptional programs linking mucosal immunity to systemic insulin resistance.

### 2.5. Prediction of Binding Sites and Molecular Docking

**Figure 8:**
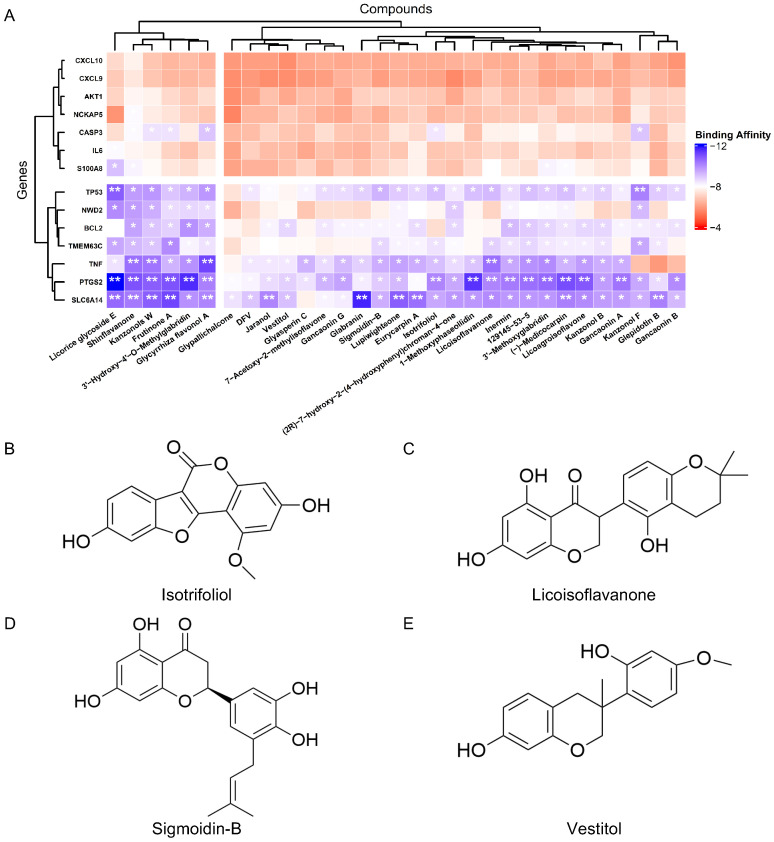
(**A**) Binding affinity heatmap of flavonoid-related small molecules docked with the 14 prioritized hub targets. * indicates values between −10 and −8; ** indicates values less than −10. (**B**–**E**) Chemical structures of the representative flavonoid-related compounds: Isotrifoliol, Licoisoflavanone, Sigmoidin-B, and Vestitol.

Seven candidate targets were initially prioritized using three topological algorithms (MNC, MCC, and EPC) in Cytoscape. Subsequent integration with seven co-dysregulated genes (common upregulated/downregulated targets) identified from GEO datasets revealed 14 hub targets through comprehensive intersection analysis. Molecular docking was systematically performed with all flavonoid-related small molecules against these prioritized targets, with results visualized in a binding affinity heatmap (Figure 8A).

**Figure 9:**
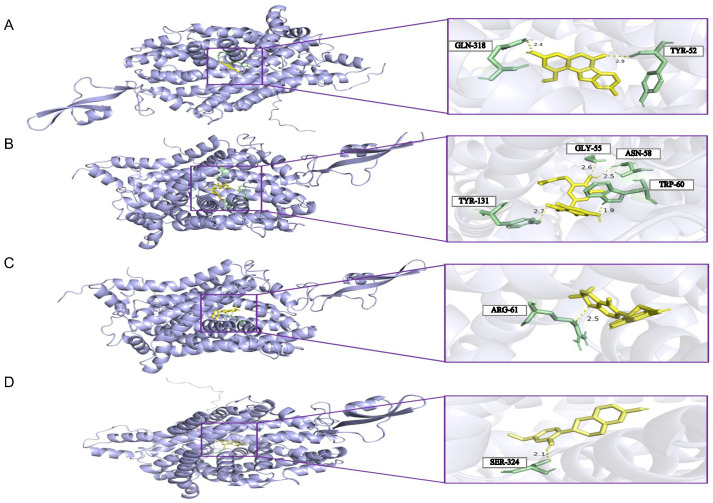
Docking results of four small molecule components in complex with SLC6A14: (**A**) Isotrifoliol; (**B**) Licoisoflavanon; (**C**) Sigmoidin-B; (**D**) Vestitol.

The molecular docking analysis focused on flavonoid constituents of SLBZS without prior experimental validation. Docking across the prioritized hub targets revealed robust multi-target engagement (e.g., Shinflavanone with TNF/PTGS2/SLC6A14; Kanzonols W with TNF/TP53/PTGS2/NWD2/SLC6A14), with PTGS2 behaving as a promiscuous hub. Critically, SLC6A14 consistently exhibited the most favorable binding energies to flavonoid ligands, thereby emerging as the primary target of interest. Guided by the previously established ADMET filter, four representative flavonoids—Isotrifoliol, Licoisoflavanone, Sigmoidin-B, and Vestitol—were retained (Figure 8B–E). Complexes between SLC6A14 and these four small molecules were then subjected to all-atom molecular dynamics simulations; Figure 9 illustrates the docking poses.

### 2.6. Molecular Dynamics Simulation Validation

**Figure 10:**
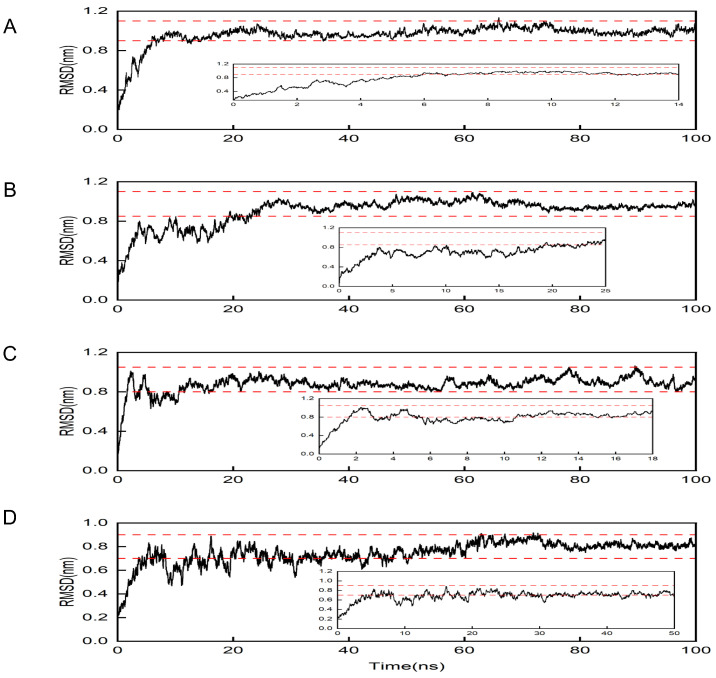
RMSD analysis of four small molecule components in complex with SLC6A14: (**A**) Isotrifoliol; (**B**) Licoisoflavanon; (**C**) Sigmoidin-B; (**D**) Vestitol.

To validate the molecular docking predictions, 100 ns all-atom molecular dynamics (MD) simulations were conducted for four high-priority complexes: isotrifoliol–SLC6A14, licoisoflavanone–SLC6A14, sigmoidin-B–SLC6A14, and vestitol–SLC6A14. Root mean square deviation (RMSD) trajectories stabilized below 0.3 nm after 20 ns equilibration, confirming structural integrity (Figure 10).

**Figure 11:**
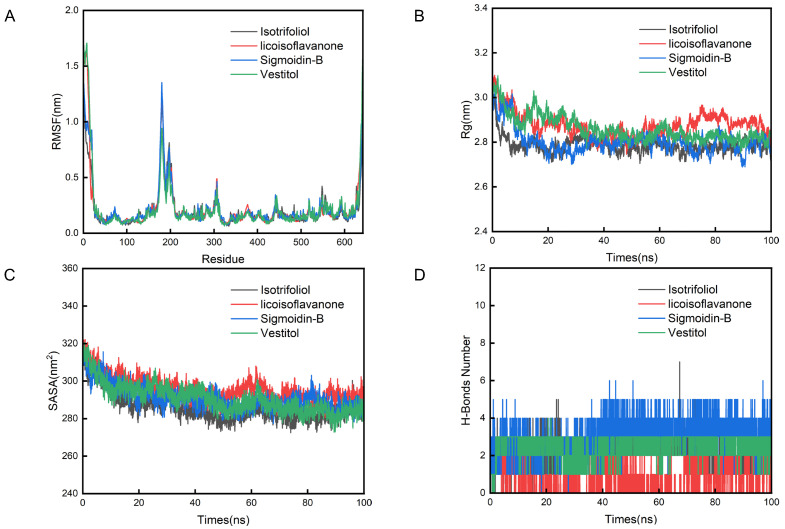
Analysis results of molecular dynamics simulations. (**A**) RMSF analysis; (**B**) Rg analysis; (**C**) SASA analysis; (**D**) hydrogen bond analysis.

The root mean square fluctuation (RMSF) analysis revealed restricted residue mobility (<1.5 nm) across all systems, particularly within the binding pocket region (residues 170–210), indicating minimal conformational perturbation during ligand accommodation (Figure 11A). The radius of gyration (Rg) profiles maintained constant compactness (2.8±0.1 nm), demonstrating ligand-induced structural stabilization (Figure 11B). The solvent accessible surface area (SASA) analysis showed consistent exposure patterns (200 ± 15 nm^2^), confirming preserved tertiary folding (Figure 11C). Hydrogen bond persistence analysis revealed differential binding modes: licoisoflavanone–SLC6A14 maintained 1.2±0.3 bonds, whereas isotrifoliol–SLC6A14, sigmoidin-B–SLC6A14, and vestitol–SLC6A14 complexes sustained 3.4±0.5 bonds throughout simulations (Figure 11D). These findings collectively validate the structural stability and target engagement specificity predicted by molecular docking.

## 3. Disscusion

The past decade has seen a growing interest in natural products and their derivatives due to their multi-target and multi-pathway regulatory advantages in complex diseases such as type 2 diabetes (T2D) and ulcerative colitis (UC). For example, indirubin derivatives have been developed as DPP-4 inhibitors for T2D treatment [17], while shikonin has shown significant anti-inflammatory effects in colitis models [18]. The integration of high-throughput screening and network pharmacology has further facilitated the identification of bioactive molecules and potential targets [19,20]. In this study, we focused on flavonoid components from the traditional Chinese formula Shen-Ling-Bai-Zhu-San (SLBZS). Using ADMET filtering and network analysis, we identified four promising compounds—Isotrifoliol, Icoisoflavanone, Sigmoidin-B, and Vestitol—along with several potential key targets.

Among these targets, SLC6A14 emerged as a novel, highly disease-relevant transporter. As a Na^+^/Cl^−^-dependent neutral amino acid transporter, SLC6A14 plays a critical role in regulating amino acid absorption and energy metabolism, with expression significantly influenced by high-fat diets [21]. Moreover, it plays an important role in immune response regulation and cell death. Under inflammatory conditions, SLC6A14 modulates amino acid availability, affecting epithelial apoptosis and immune responses [22]. In T2D treatment, SLC6A14 has also shown potential. A study found that the commonly used drug Metformin (MET) significantly altered the expression of SLC6A14 in the liver of diet-induced obese (DIO) mice, correlating with improvements in insulin sensitivity. These changes were associated with improvements in glucose absorption and amino acid transport, which may contribute to better metabolic regulation in obesity and insulin resistance [23]. Further research indicated that SNPs in the SLC6A14 gene were linked to obesity, particularly in obese males, suggesting a gender-specific effect. This further solidifies its potential as a therapeutic target for obesity [24]. SLC6A14’s role in UC has also garnered attention. It was found to be significantly upregulated in the intestinal tissues of UC patients, with expression correlating with UC activity. Immune cell infiltration analysis revealed strong associations between SLC6A14 and various immune cell types, further supporting its role in UC’s immune response [25]. Additionally, SLC6A14 regulates the NLRP3 inflammasome and promotes pyroptosis (a form of inflammatory cell death) in UC. Reducing SLC6A14 expression was found to suppress pyroptosis in UC models, highlighting its potential in modulating UC-associated inflammation [26]. In our docking and molecular dynamics simulations, both Isotrifoliol and Sigmoidin-B showed favorable binding stabilities with SLC6A14, further underscoring its potential as a dual-purpose therapeutic target. These findings support SLC6A14 as a promising target for combined T2D and UC treatment.

In addition to SLC6A14, our study identified several conventional inflammation- and metabolism-associated targets, including IL6, TNF, AKT1, CASP3, BCL2, CXCL10, CXCL9, and S100A8. These molecules share common roles in the pathological mechanisms of both T2D and UC. IL-6, for instance, modulates inflammation and epithelial homeostasis through the STAT3 pathway [27], and is closely related to insulin resistance [28,29]. TNF-α, a classic pro-inflammatory cytokine, is a validated therapeutic target in UC and contributes to chronic low-grade inflammation in T2D [30,31]. AKT1 serves as a key regulator across inflammation, metabolism, and apoptosis, making it a promising target in dual-disease contexts [32]. Chemokines like CXCL10 and CXCL9 mediate immune cell recruitment and have been implicated in beta-cell dysfunction in diabetes and immune responses in UC [33,34,35].

GO and KEGG enrichment analyses identified biological processes potentially pivotal in regulating immune-related cell adhesion, proliferation, and pathogen responses, indicating a close connection with inflammation, immunity, and the tumor microenvironment. Additionally, several key pathways, including MAPK, PI3K-Akt, apoptosis, and TNF signaling, are central to the onset and progression of both T2D and UC. These pathways significantly impact chronic inflammation and the development of complications in both diseases by regulating cellular metabolism, immune responses, apoptosis, and vascular function. The PI3K/Akt signaling pathway is a critical component of insulin signaling, playing an essential role in regulating glucose metabolism and insulin resistance [36]. Upon insulin binding to its receptor, PI3K is activated, which subsequently activates Akt. Activated Akt plays a crucial role in cellular processes such as glucose transport, glycogen synthesis, cell proliferation, and survival [37]. Multiple studies have shown that modulating the PI3K/Akt pathway can improve the condition of T2D. For instance, the metabolic products of certain traditional Chinese medicines have been shown to exert hypoglycemic effects by targeting the PI3K/Akt pathway [38]. Additionally, specific dietary components and lifestyle changes, such as exercise, may protect pancreatic β-cells by modulating the PI3K/Akt pathway, offering potential therapeutic benefits for T2D [39]. Research has indicated that the PI3K/Akt pathway is overactivated in the colon tissues of UC patients, playing a role in regulating inflammatory responses [40]. Traditional Chinese medicines, including Sanhuang decoction enema, have been shown to treat UC by modulating various signaling pathways, such as the PI3K/Akt pathway [41]. The MAPK (mitogen-activated protein kinase) signaling pathway is a highly conserved pathway that regulates key processes including cell proliferation, differentiation, apoptosis, immune responses, and stress responses [42]. In T2D, abnormal activation of the MAPK pathway is closely linked to insulin resistance, β-cell dysfunction, and the development of diabetes-related complications. Specifically, prolonged exposure to high concentrations of saturated fatty acids can induce apoptosis of pancreatic β-cells through the activation of the p38 MAPK pathway, leading to β-cell dysfunction and contributing to the onset of T2D [43]. The TCF7L2 gene plays a role in the onset of diabetes by inhibiting pancreatic β-cell dedifferentiation via the ERK/MAPK signaling pathway [44]. Furthermore, various studies have demonstrated that inhibiting the MAPK pathway can alleviate UC symptoms. For instance, Fraxin alleviates UC by modulating the MAPK pathway [45]; Shionone relieves UC by regulating the p38 MAPK/NF-κB pathway and 7-hydroxycoumarin improves UC in mice by inhibiting the MAPK pathway [46]. These studies suggest that the combined treatment of T2D and UC with SLBZS involves a synergistic modulation of multiple pathways. These crucial pathways represent potential targets for future research aimed at treating T2D and UC, with a focus on regulating insulin resistance, immune responses, and modulating cell proliferation and metabolism. Our findings contribute to the development of novel therapeutic strategies targeting both diseases.

However, this study has several limitations. First, as an in silico investigation, our findings remain predictive and require subsequent wet-lab validation. Second, the molecular dynamics simulations were conducted only on one prioritized target (SLC6A14), which may not fully capture the broader spectrum of transporter or protein interactions. Third, our analyses mainly focused on flavonoids, while other chemical classes present in SLBZS—such as saponins, polysaccharides, and alkaloids—were underrepresented. Future work will expand both target and chemotype coverage and pursue systematic experimental validation to strengthen the translational relevance of these results.

In conclusion, through an integrative strategy combining ADMET screening, network pharmacology, target prediction, and molecular simulations, we systematically elucidated the potential mechanisms of SLBZS flavonoids in T2D and UC comorbidity. Among the findings, SLC6A14 stands out as a promising, underexplored target with significant implications for dual-disease intervention. This discovery provides a novel perspective on the shared pathophysiology of T2D and UC, offering theoretical support for SLC6A14-targeted natural product drug development. Nevertheless, further experimental validations, including in vitro and in vivo models, are essential to confirm the biological activity and mechanistic roles of these flavonoid compounds.

## 4. Materials and Methods

### 4.1. Bioactive Constituent Screening and Target Profiling

The deca-herbal composition of SLBZS was subjected to systematic target identification through multi-database interrogation. The constituent bioactivity data were retrieved primarily from the pharmacology database of Traditional Chinese Medicine Systems (TCMSP, version 2.3). Canonical SMILES notation of prioritized phytochemicals was acquired via PubChem [47], followed by target prediction using SwissTargetPrediction [48] with probability score >0.7. Complementary target annotation was performed through Comparative Toxicogenomics Database [49] mining. For constituent prioritization, evidence-based stratification was employed, which included experimentally validated compounds from the peer-reviewed literature, as well as pharmaceutically prevalent chemotypes. These compounds were further subjected to molecular docking and ADMET profiling. The pkCSM platform [50] was used to facilitate quantitative drug-likeness assessment through the following steps:

- Structural filters: Lipinski’s [51] rules (molecular weight, MW ≤ 500 Da; octanol-water partition coefficient, LogP ≤ 5; hydrogen bond donors, HBD ≤ 5; hydrogen bond acceptors, HBA ≤ 10) and Veber’s [52] rules (rotatable bonds ≤ 10; topological polar surface area, TPSA ≤ 140 ${\text{Å}}^{2}$).
- Pharmacokinetic parameters: Caco-2 [53] (quantitative prediction of Caco-2 permeability, QPPCaco > 0.9); steady-state volume of distribution (VDss ≥ 0.45 L/kg).
- Metabolic stability: Cytochrome P450 3A4 (CYP3A4) substrate probability [54] (TopBank score ≥ 0.8); total clearance (CLtot < 5 mL/min/kg).
- Toxicological thresholds: Drug-induced liver injury (DILI) prediction [55] via validated random forest classifiers.

Following the above procedures, a total of 173 unique compounds were obtained from the TCMSP database after deduplication, corresponding to the 10 constituent herbs of SLBZS. Literature validation further refined this set to 103 compounds that lacked clinical evidence or prior experimental investigation in the context of the two target diseases. Structural classification of these 103 compounds revealed that flavonoids constituted the largest chemical category, with 41 members. Subsequent ADMET evaluation and comparative screening based on predefined criteria ultimately yielded four small molecules as the final candidate active constituents.

### 4.2. Identification of Potential Targets for Treating Both Diseases

Transcriptomic datasets for type 2 diabetes (T2D) and ulcerative colitis (UC) were retrieved from GEO. For T2D, we used GSE23343 (liver biopsies from 10 T2D patients and 7 normal glucose-tolerant subjects) and GSE76894 (206 pancreatic islet samples, including 55 T2D and 116 non-diabetic controls from organ donors and pancreatectomy patients). For UC, we used GSE87466 (colonic mucosal biopsies from 87 UC patients and 21 normal subjects) and GSE107499 (paired colonic biopsies from inflamed and non-inflamed regions of UC patients). These datasets provide human tissue-derived transcriptomic profiles relevant to metabolic and inflammatory dysfunction.

For each disease, differentially expressed genes (DEGs) were identified using the thresholds |log_2_FC| > 0.5 and adjusted p<0.05. Overlapping DEGs between T2D and UC were considered primary candidate targets. In parallel, disease-associated genes were retrieved from GeneCards for both T2D and UC and intersected with SLBZS-predicted targets. Finally, shared therapeutic targets were determined by the overlap of SLBZS-related targets, GEO-derived DEGs, and GeneCards disease genes.

### 4.3. Protein–Protein Interaction Network Construction

To elucidate the multi-target mechanisms of SLBZS in T2D-UC comorbidity, a protein–protein interaction (PPI) network was reconstructed using the STRING database (version 12.0) with an interaction confidence threshold > 0.6 (medium confidence). Network topology analysis was conducted in Cytoscape (version 3.9.1). First, nodes with betweenness centrality > 0.2 were retained to ensure that only proteins with important bridging roles in the network were considered. Subsequently, three complementary node-ranking algorithms were applied: Maximum Neighborhood Component (MNC), which emphasizes local connectivity within dense clusters; Maximal Clique Centrality (MCC), which identifies nodes participating in multiple maximal cliques, highlighting tightly connected substructures; and Edge Percolated Component (EPC), which captures node robustness under edge removal, reflecting network resilience. For each algorithm, the top 10 ranked nodes were extracted, and nodes consistently prioritized across methods were designated as core therapeutic targets. This two-step strategy ensured that the final core targets were both structurally central and functionally influential in the PPI network.

### 4.4. Pathway Analysis

To elucidate the system-level therapeutic mechanisms of SLBZS in T2D-UC comorbidity, we integrated multidimensional pathway analytics as follows:

- KEGG/GO enrichment analysis identified significantly enriched pathways (adjusted p<0.05) among formula–disease overlapping targets.
- Contextualized pathway prioritization was achieved through PROGENy-derived weighting of 14 therapeutic pathways, where target-specific coefficients were calibrated against herb–component interaction gradients.
- Pathway activity inference was implemented using decoupleR’s multi-linear model (MLM), which projects gene-level differential expression ($\log_{2}\mathrm{FC}$) onto curated pathway networks. Formally, pathway activity scores S are computed via:
  (1) $$S={\left(W^{⊤}W\right)}^{-1}W^{⊤}y$$
  where $y\in R^{n}$ is the vector of observed log fold changes for *n* genes, and $W\in R^{n\times p}$ is the PROGENy-derived weight matrix mapping genes to *p* pathways.
- The resulting scores S were subsequently z-normalized to enable cross-sample comparison:
  (2) $${\hat{S}}_{j}=\frac{S_{j}-\mu_{j}}{\sigma_{j}},\;\forall j\in \left\{1,\ldots ,p\right\}$$
  where $\mu_{j}$ and $\sigma_{j}$ denote the mean and standard deviation of $S_{j}$ across all experimental conditions. These normalized scores were visualized as clustered heatmaps and ranked bar plots.
- For mechanistic dissection of four key pathways—JAK–STAT, PI3K, TNFα, and Androgen—we defined a directional concordance index for each pathway gene *g*:
  (3) $$C_{g}=\mathrm{sign}\left(w_{g}\right)\cdot \mathrm{sign}\left(\log_{2}{\mathrm{FC}}_{g}\right)$$
  where $w_{g}$ is the PROGENy-derived regulatory weight and $\log_{2}{\mathrm{FC}}_{g}$ the observed expression fold-change. Genes with $C_{g}=+1$ indicate concordant regulation, whereas $C_{g}=-1$ indicate discordant or counter-regulatory behavior. These interactions were displayed in weighted scatter plots annotated with gene symbols.

This triaxial computational framework systematically delineates the therapeutic mechanism of SLBZS in the context of T2D–UC comorbidity. By orchestrating the crosstalk between metabolic and inflammatory pathways, the model captures the core regulatory dynamics underlying treatment effects. Notably, the predicted pathway activities exhibit strong concordance with transcriptomic profiles observed under disease conditions, supporting the biological validity and translational relevance of the approach.

### 4.5. Molecular Docking

The molecular docking simulations were conducted to predict ligand-target interactions using a detailed protocol [56]. Initially, 2D structures of bioactive constituents from SLBZS were retrieved from PubChem (https://pubchem.ncbi.nlm.nih.gov) and subjected to conformational energy minimization in Chem3D using the MMFF94 force field. The target protein structures were then processed in PyMOL, where solvent molecules and co-crystallized ligands were removed (command: remove solvent; remove organic). For the docking simulations, AutoDock Vina 1.2.3 was used with cubic grids centered on the predicted binding sites; the grid edge length was 25 Å (size_x = size_y = size_z = 25 Å). generating 10 binding poses per ligand, ranked by binding affinity (kcal/mol). The binding free energy thresholds were set at ≤−5 kcal/mol (moderate) and ≤−8 kcal/mol (high-affinity) for pharmacological relevance assessment.

### 4.6. Molecular Dynamics Simulation Analysis

Molecular dynamics (MD) simulations were conducted using GROMACS 2023.2 to assess the conformational stability and dynamic behavior of the ligand-protein complexes identified through docking [57]. Ligand topologies were generated with electrostatic potential charges integrated with the GAFF2 force field through AmberTools14 and ACPYPE [58], while the AMBER14SB force field parameterized the protein. The solvated system was constructed within a cubic periodic boundary using the SPC water model, with Na^+^/Cl^−^ counterions neutralizing system charges. Following energy minimization via the steepest descent algorithm (convergence criterion: Fmax < 1000 kJ/(mol·nm^2^)), a two-phase equilibration protocol was implemented: initial 100 ps NVT ensemble with 1000 kJ/(mol·nm^2^) positional restraints on the ligand, succeeded by 100 ps NPT ensemble without restraints. Production simulations extended to 100 ns under NPT conditions (2 fs timestep), employing LINCS constraints for hydrogen bonds [59] and Particle Mesh Ewald (PME) [60] for long-range electrostatics. Trajectory analyses quantified backbone root-mean-square deviation (RMSD), radius of gyration (Rg), residue-specific root-mean-square fluctuation (RMSF), solvent-accessible surface area (SASA), and persistent hydrogen bond interactions using native GROMACS utilities, complemented by energy convergence profiles and thermodynamic parameter monitoring to validate system equilibration.

### 4.7. Software and Parameters

In this study, several software tools and databases were used. The bioactivity data were retrieved from the Traditional Chinese Medicine Systems Pharmacology Database (TCMSP, version 2.3). Target prediction was performed using SwissTargetPrediction (http://www.swisstargetprediction.ch) with a probability score >0.7. Protein–protein interaction (PPI) networks were constructed using STRING (version 12.0) with an interaction confidence threshold >0.6. Network topology analysis was conducted using Cytoscape (version 3.9.1) with centrality algorithms, including Maximum Neighborhood Component (MNC), Maximal Clique Centrality (MCC), Edge Percolated Component (EPC), and Degree Centrality. Molecular docking simulations were carried out using AutoDock Vina (v1.2.3) with cubic grid boxes (25 Å per side) centered on the predicted binding pockets; binding-affinity thresholds were set to $\leq -5\;\mathrm{kcal},{\mathrm{mol}}^{-1}$ (moderate) and $\leq -8\;\mathrm{kcal},{\mathrm{mol}}^{-1}$ (high). Molecular dynamics simulations were conducted using GROMACS (version 2023.2), with the AMBER14SB force field for proteins, GAFF2 for ligands, and the SPC water model. The simulation was performed for 100 ns with a time step of 2 fs, employing LINCS constraints for hydrogen bonds and Particle Mesh Ewald (PME) for long-range electrostatics.

## 5. Conclusions

This study revealed the multimodal mechanisms of SLBZS in managing T2D–UC comorbidity through integrative multi-omics analysis and molecular validation. Key flavonoid constituents demonstrated stable and pharmacologically relevant interactions with hub targets, with SLC6A14 emerging as a central regulator of immunometabolic crosstalk. These findings provide mechanistic support for the traditional use of SLBZS and highlight its potential for broader application in inflammatory–metabolic disorders. While the reliance on computational predictions and limited in vivo validation represents a constraint, this integrative approach underscores future directions for bridging systems pharmacology and experimental research to facilitate the clinical translation of herbal medicine.

## Figures and Tables

**Table 1:** Physicochemical and ADMET profiles of four flavonoid candidates.

Physicochemical Properties	Isotrifoliol	Licoisoflavanone	Sigmoidin-B	Vestitol
Molecular Weight (MW)	298.25	354.358	356.374	272.3
No. of Rotatable Bonds (<10)	1	1	3	2
No. of H-bond Acceptors (<10)	6	6	6	4
No. of H-bond Donors (<5)	2	3	4	2
LogP (<5)	3.112	3.347	3.724	2.825
LogS (Water Solubility)	−3.67	−5.93	−5.58	−4.45
Caco-2 (logPapp) Prediction	−4.62	−5.02	−5.81	−4.74
Human Intestinal Absorption (HIA)	0.998	0.959	0.881	0.985
Human Oral Bioavailability (20%)	0.369	0.38	0.418	0.63
P-Glycoprotein Inhibitor	Non-Inhibitor	Non-Inhibitor	Non-Inhibitor	Non-Inhibitor
VDss (Human)	1.58	0.88	0.92	1.14
Blood–Brain Barrier (BBB)	−2.17	−2.99	−2.93	−2.43
CYP3A4 Substrate Probability	Non-Substrate	Non-Substrate	Non-Substrate	Non-Substrate
Clearance (mL/min/kg)	2.7	10.29	10.42	6.85
Drug-Induced Liver Injury (DILI)	Safe	Safe	Safe	Safe

## Data Availability

The Publicly available datasets were analyzed in this study. The data are openly available in the NCBI Gene Expression Omnibus (GEO) under the following accession numbers: GSE23343 https://www.ncbi.nlm.nih.gov/geo/query/acc.cgi?acc=GSE23343, GSE76894 https://www.ncbi.nlm.nih.gov/geo/query/acc.cgi?acc=GSE76894, GSE87466 https://www.ncbi.nlm.nih.gov/geo/query/acc.cgi?acc=GSE87466, and GSE107499 https://www.ncbi.nlm.nih.gov/geo/query/acc.cgi?acc=GSE107499 (accessed on 5 October 2025).
